# Supplementary figures and images for: CRP immunodeposition and proteomic analysis in abdominal aortic aneurysm
Source: PLoS One. 2021 Aug 24;16(8):e0245361. doi: 10.1371/journal.pone.0245361 (PMC8384196; doi:10.1371/journal.pone.0245361)

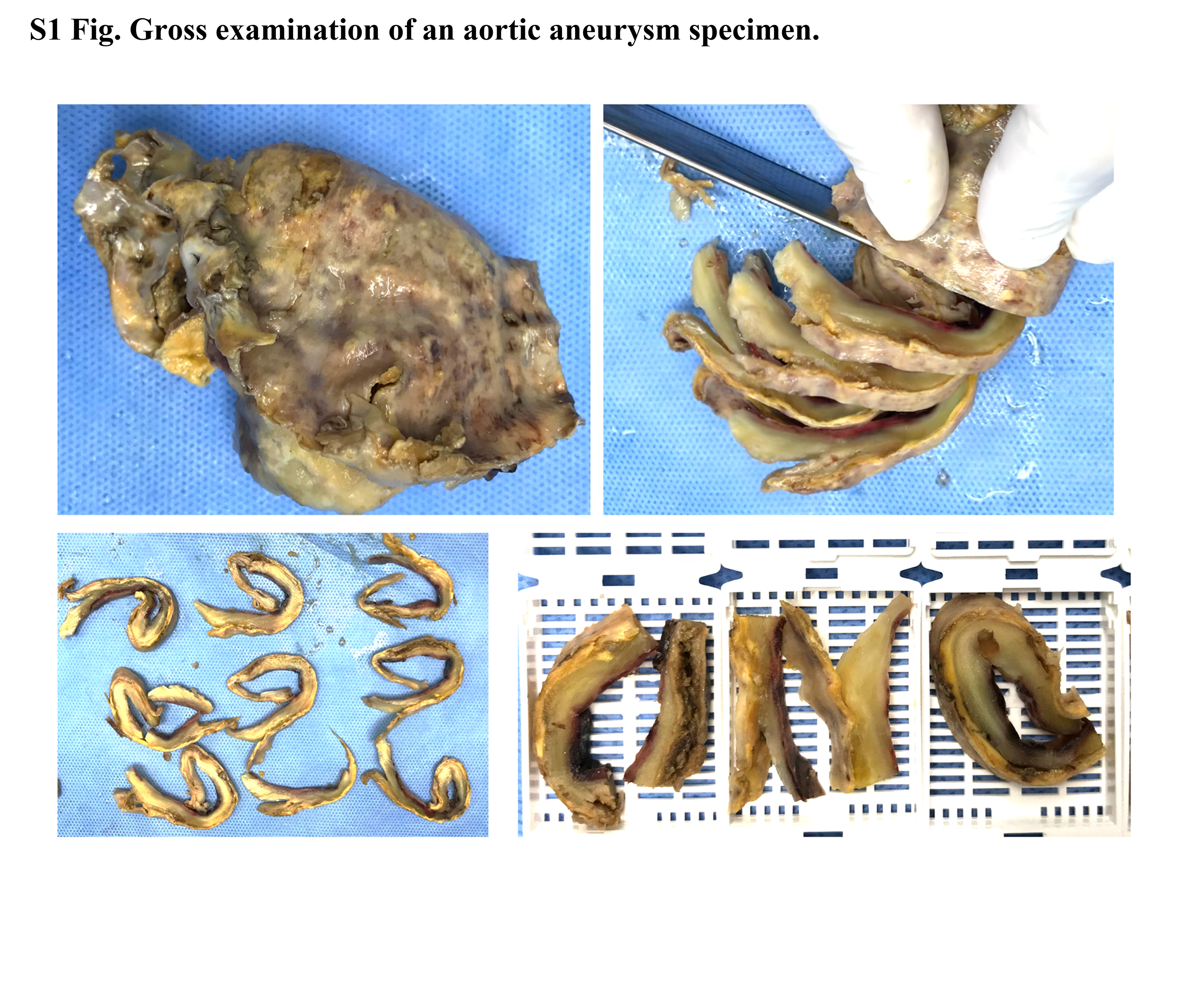

Supplement: S1 Fig — (TIF) [file pone.0245361.s001.tif]

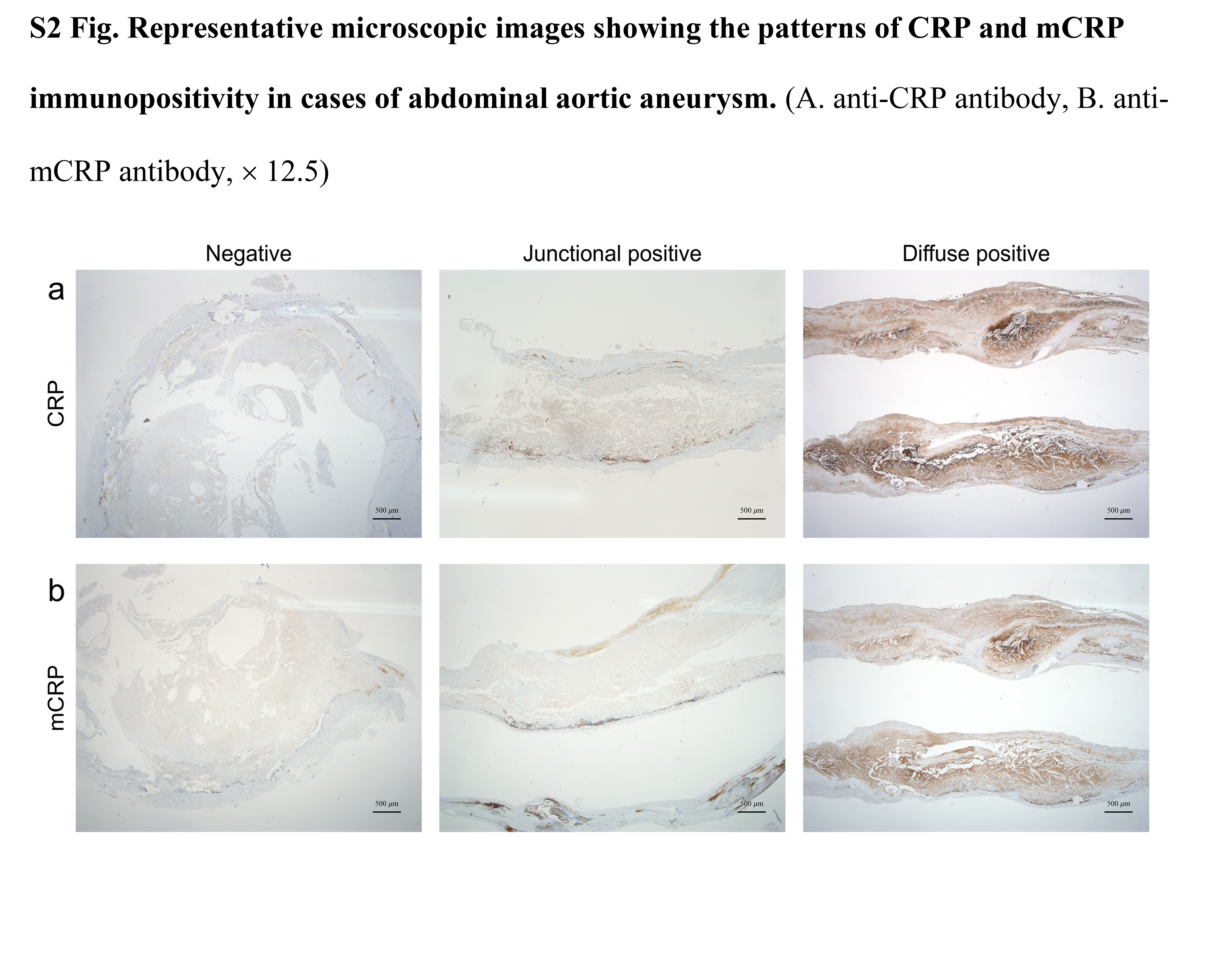

Supplement: S2 Fig — (A. anti-CRP antibody, B. anti-mCRP antibody, × 12.5). (TIF) [file pone.0245361.s002.tif]

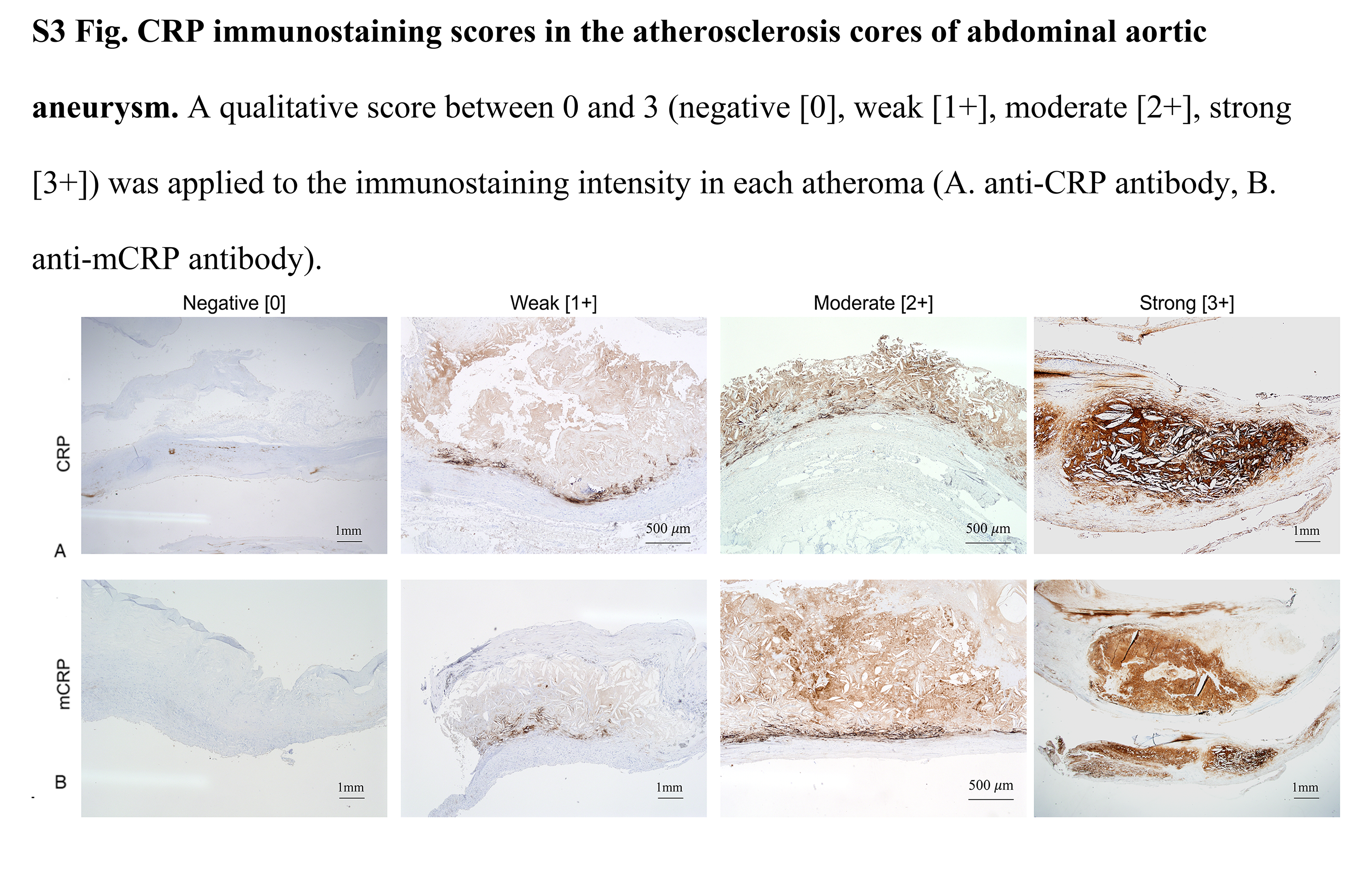

Supplement: S3 Fig — A qualitative score between 0 and 3 (negative [0], weak [1+], moderate [2+], strong [3+]) was applied to the immunostaining intensity in each atheroma (A. anti-CRP antibody, B. anti-mCRP antibody). (TIF) [file pone.0245361.s003.tif]

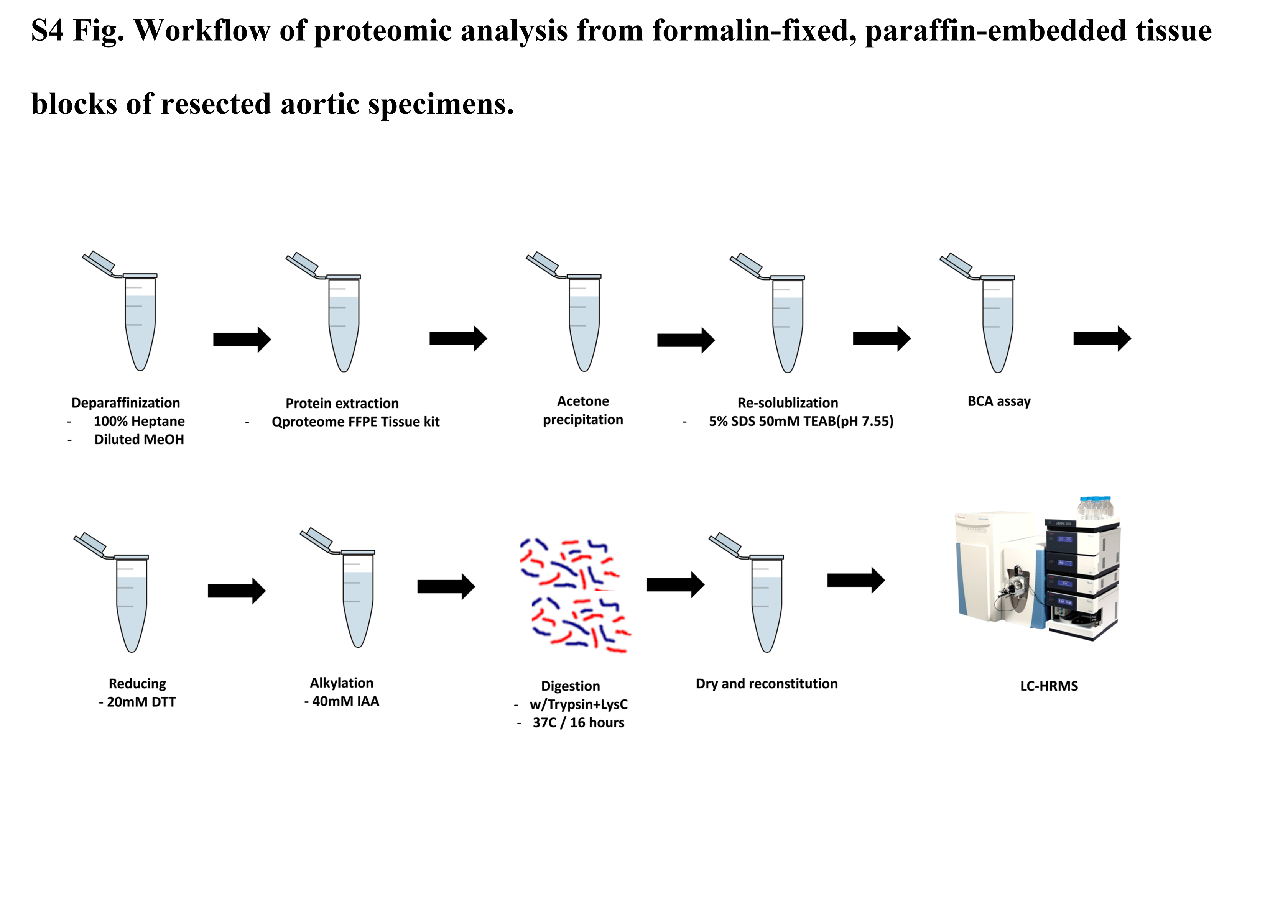

Supplement: S4 Fig — (TIF) [file pone.0245361.s004.tif]

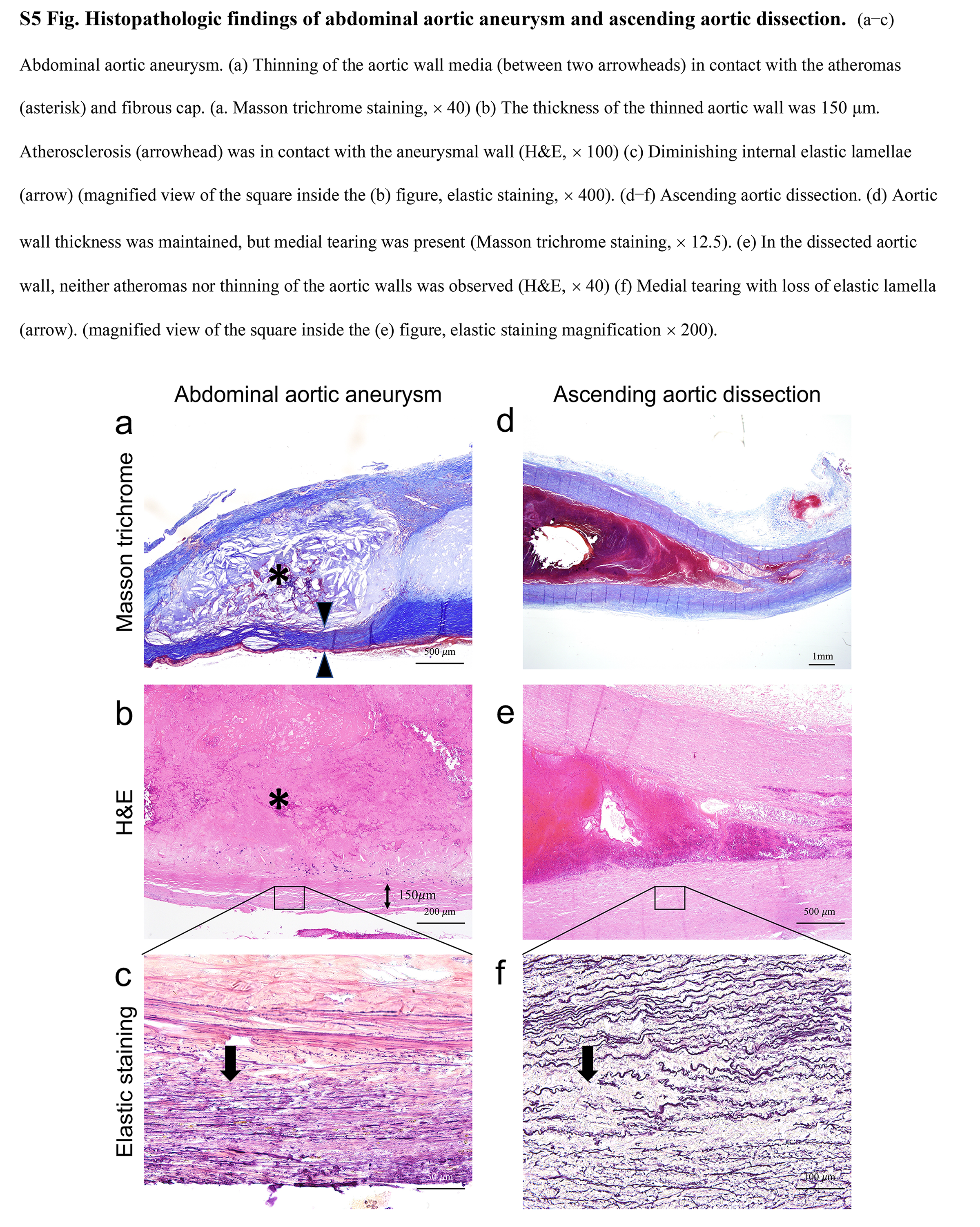

Supplement: S5 Fig — (a–c) Abdominal aortic aneurysm. (a) Thinning of the aortic wall media (between two arrowheads) in contact with the atheromas (asterisk) and fibrous cap. (a. Masson trichrome staining, × 40) (b) The thickness of the thinned aortic wall was 150 μm. Atherosclerosis (arrowhead) was in contact with the aneurysmal wall (H&E, × 100) (c) Diminishing internal elastic lamellae (arrow) (magnified view of the square inside the (b) figure, elastic staining, × 400). (d–f) Ascending aortic dissection. (d) Aortic wall thickness was maintained, but medial tearing was present (Masson trichrome staining, × 12.5). (e) In the dissected aortic wall, neither atheromas nor thinning of the aortic walls was observed (H&E, × 40) (f) Medial tearing with loss of elastic lamella (arrow). (magnified view of the square inside the (e) figure, elastic staining magnification × 200). (TIF) [file pone.0245361.s005.tif]

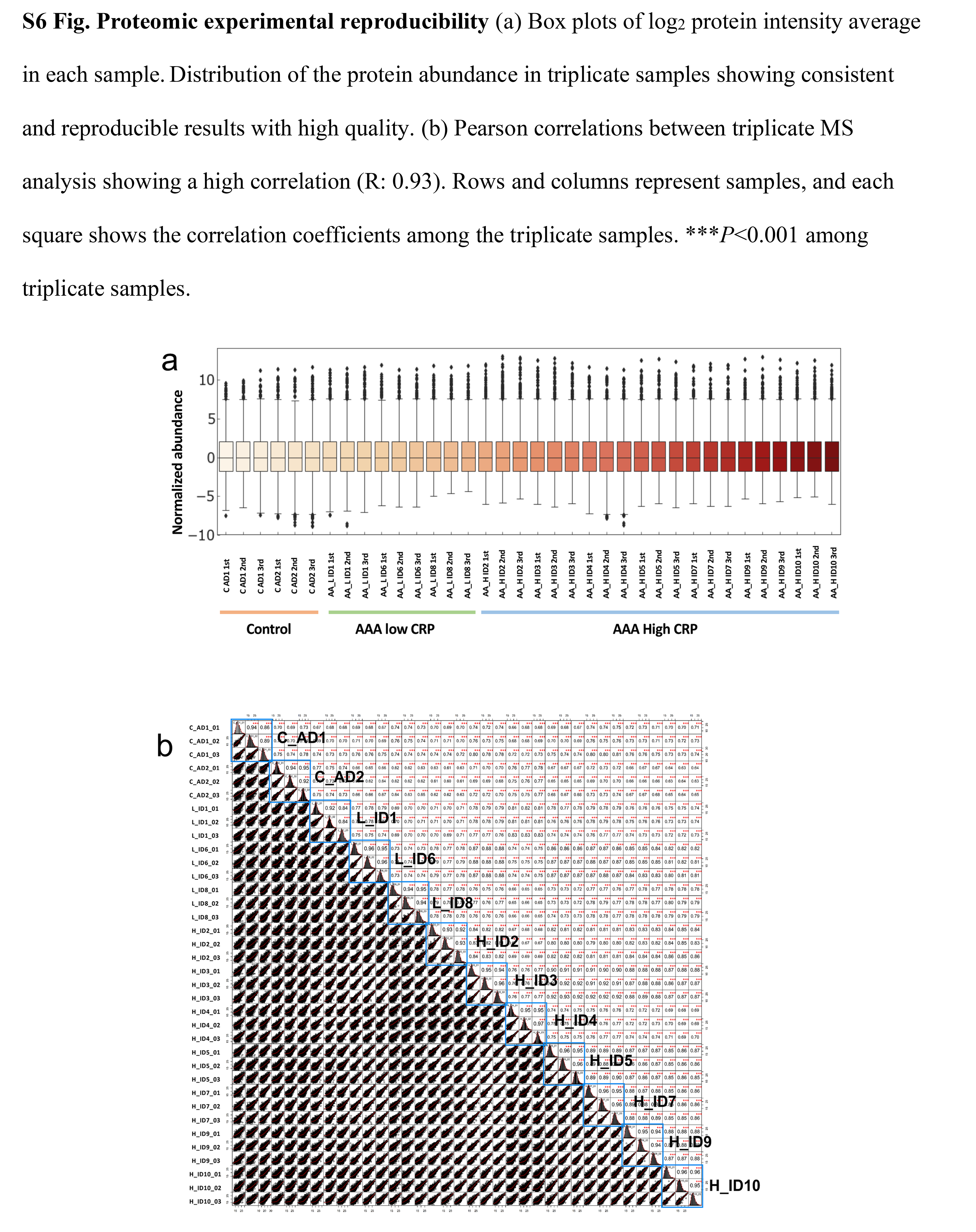

Supplement: S6 Fig — (a) Box plots of log2 protein intensity average in each sample. Distribution of the protein abundance in triplicate samples showing consistent and reproducible results with high quality. (b) Pearson correlations between triplicate MS analysis showing a high correlation (R: 0.93). Rows and columns represent samples, and each square shows the correlation coefficients among the triplicate samples. ***P<0.001 among triplicate samples. (TIF) [file pone.0245361.s006.tif]
