# Supplementary material for: CRP immunodeposition and proteomic analysis in abdominal aortic aneurysm
Source: PLoS One. 2021 Aug 24;16(8):e0245361. doi: 10.1371/journal.pone.0245361 (PMC8384196; doi:10.1371/journal.pone.0245361)

A LXR/RXR Activation

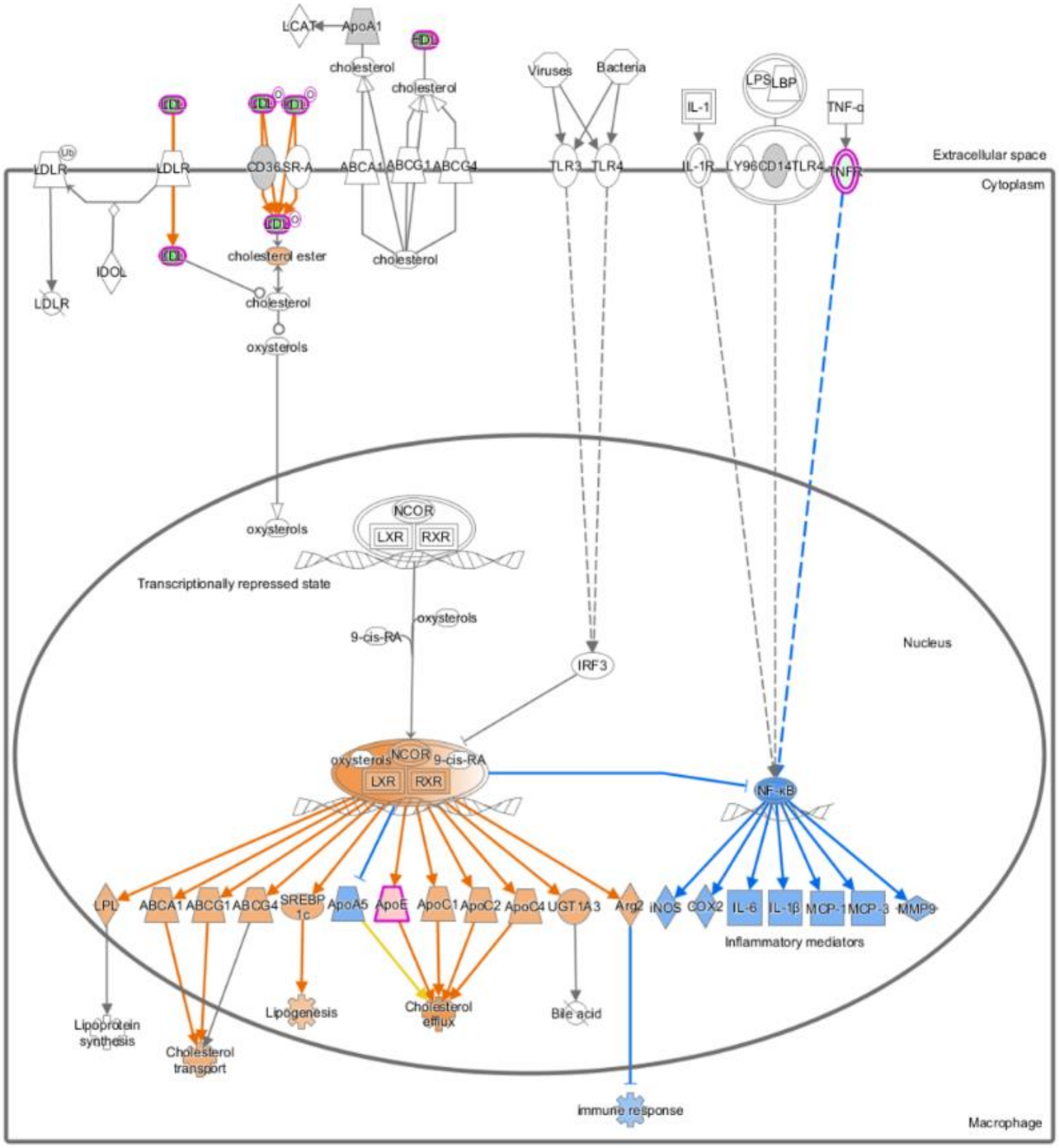

B Coagulation system

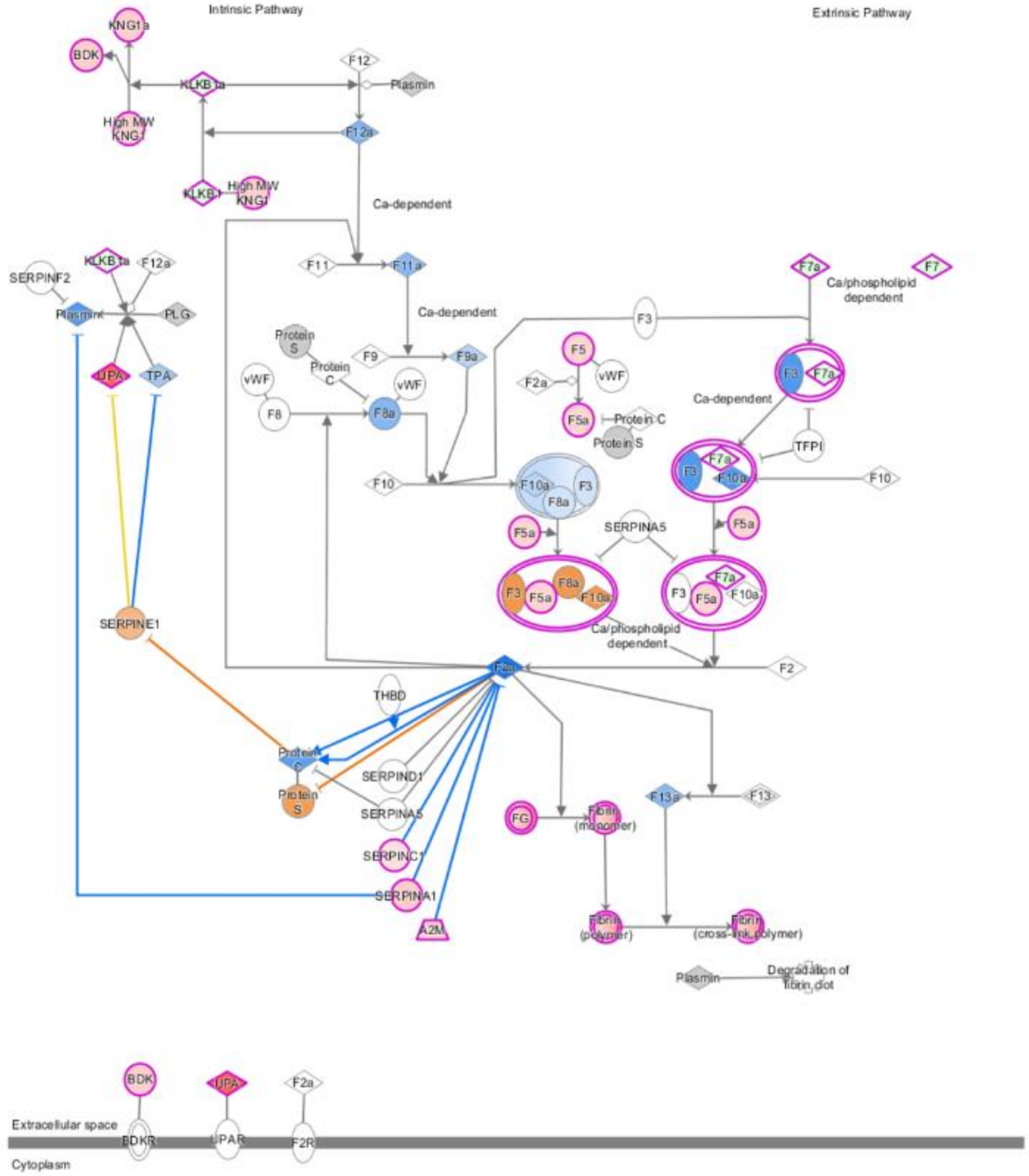

C Acute phase response signaling

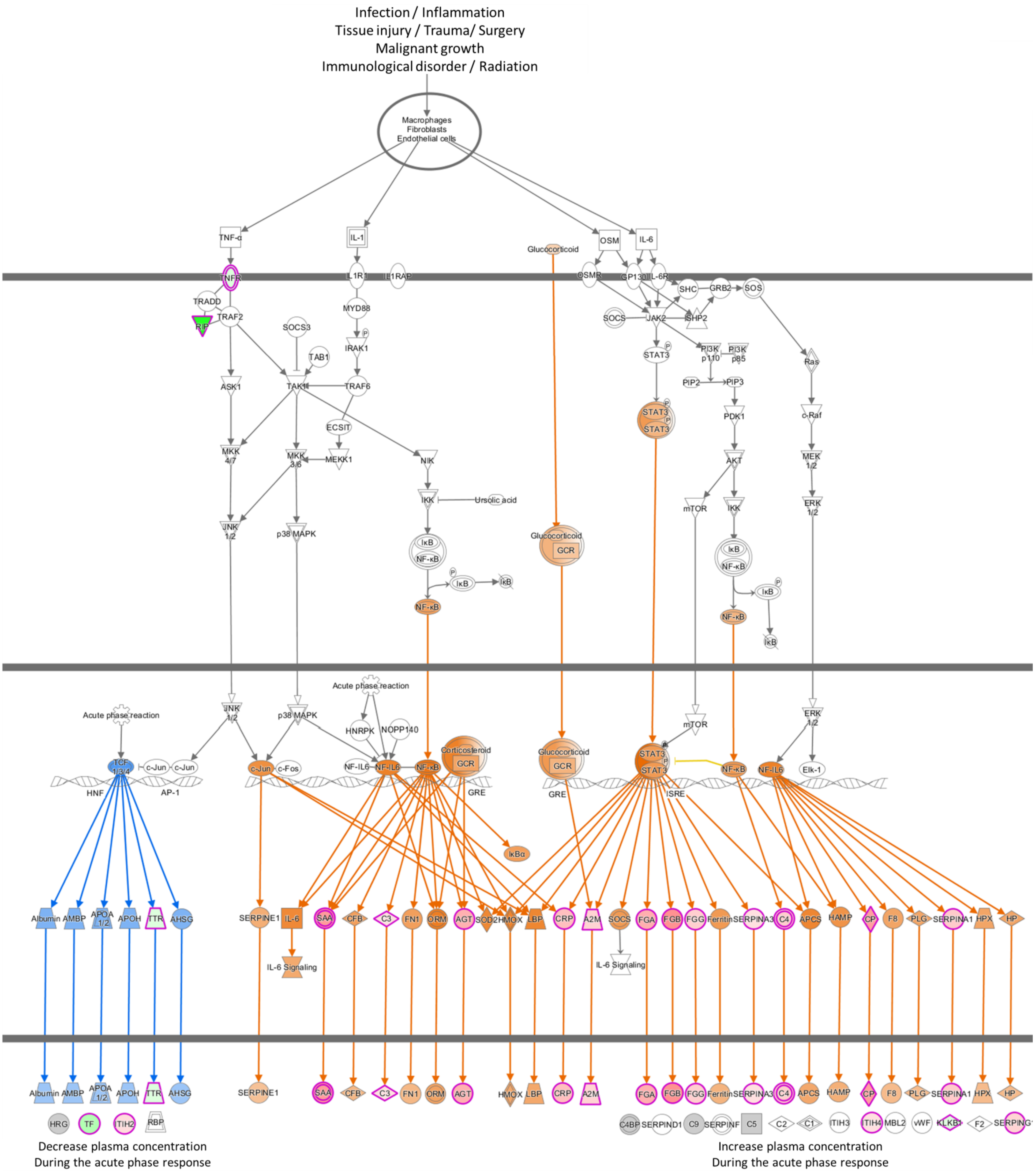

D Extrinsic prothrombin activation pathway

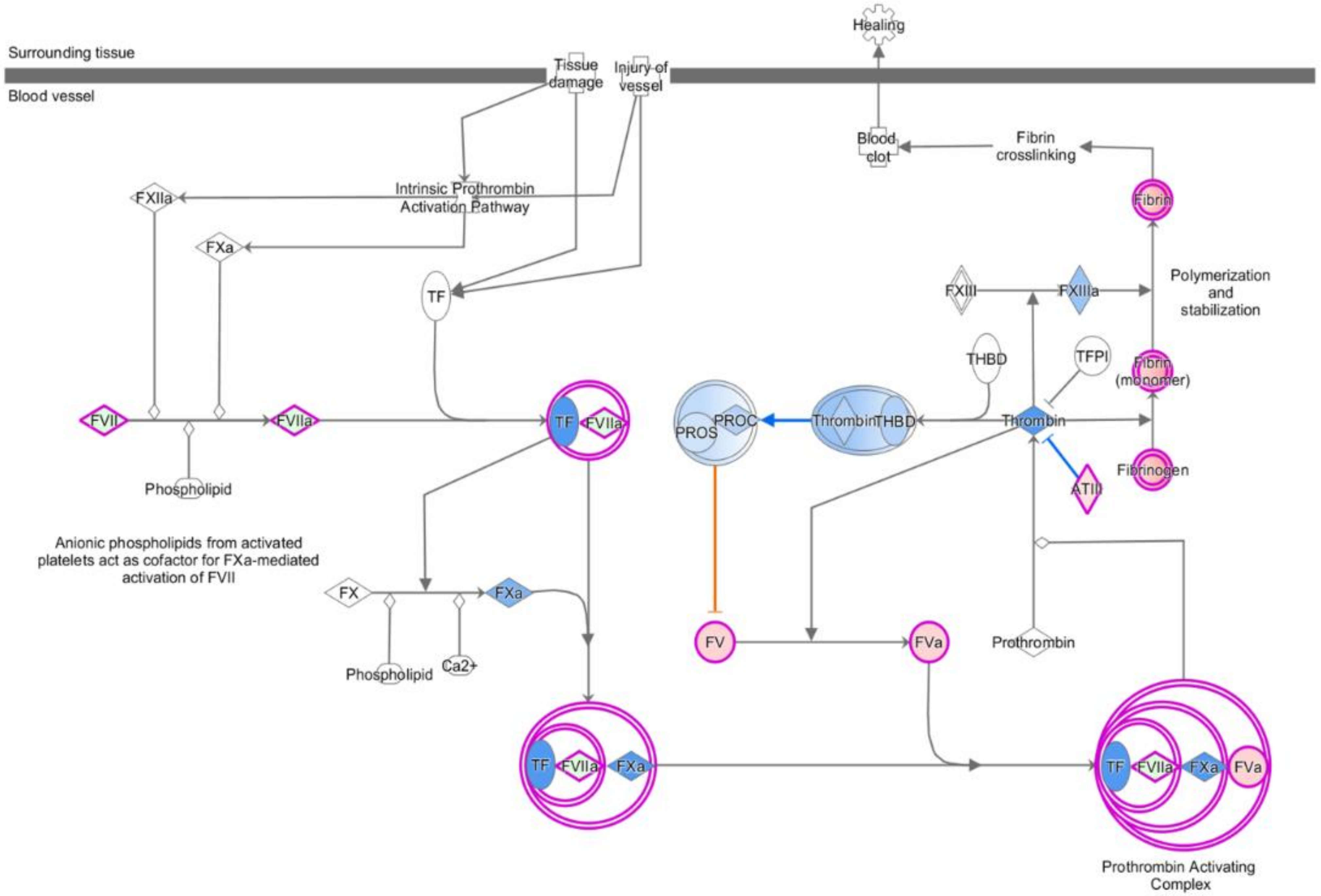

E Intrinsic prothrombin activation pathway

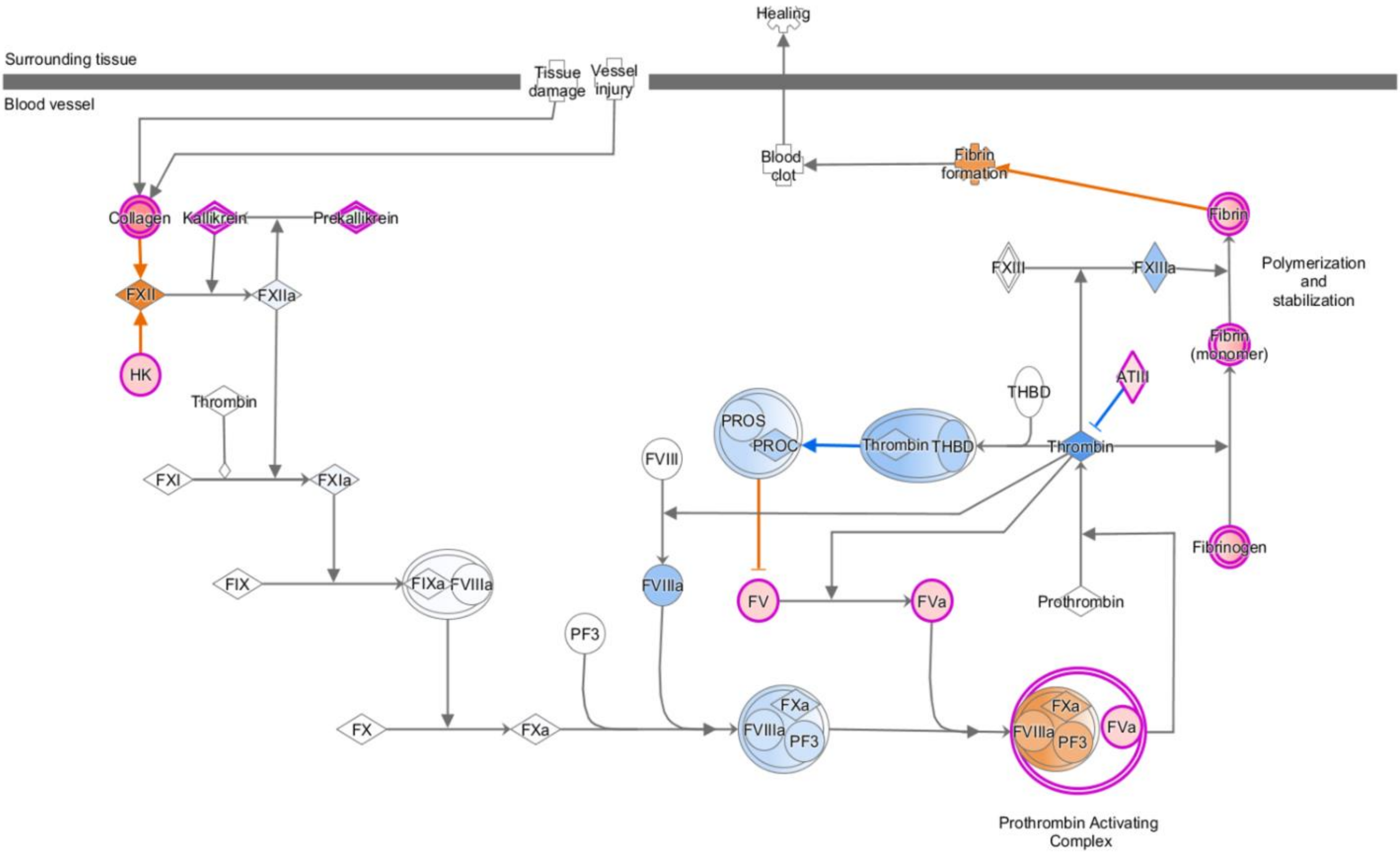

F Atherosclerosis Signaling

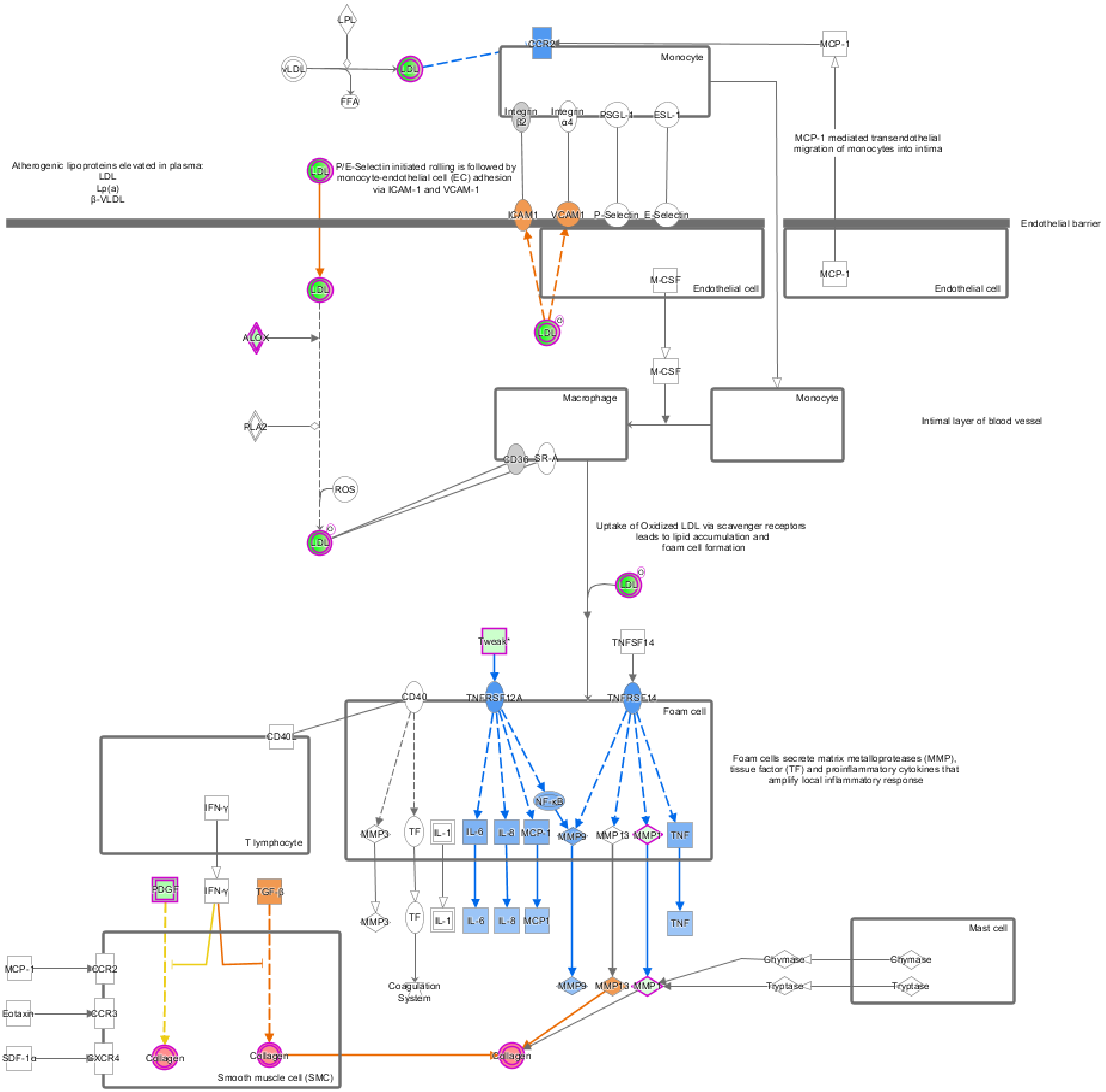

G Complement System

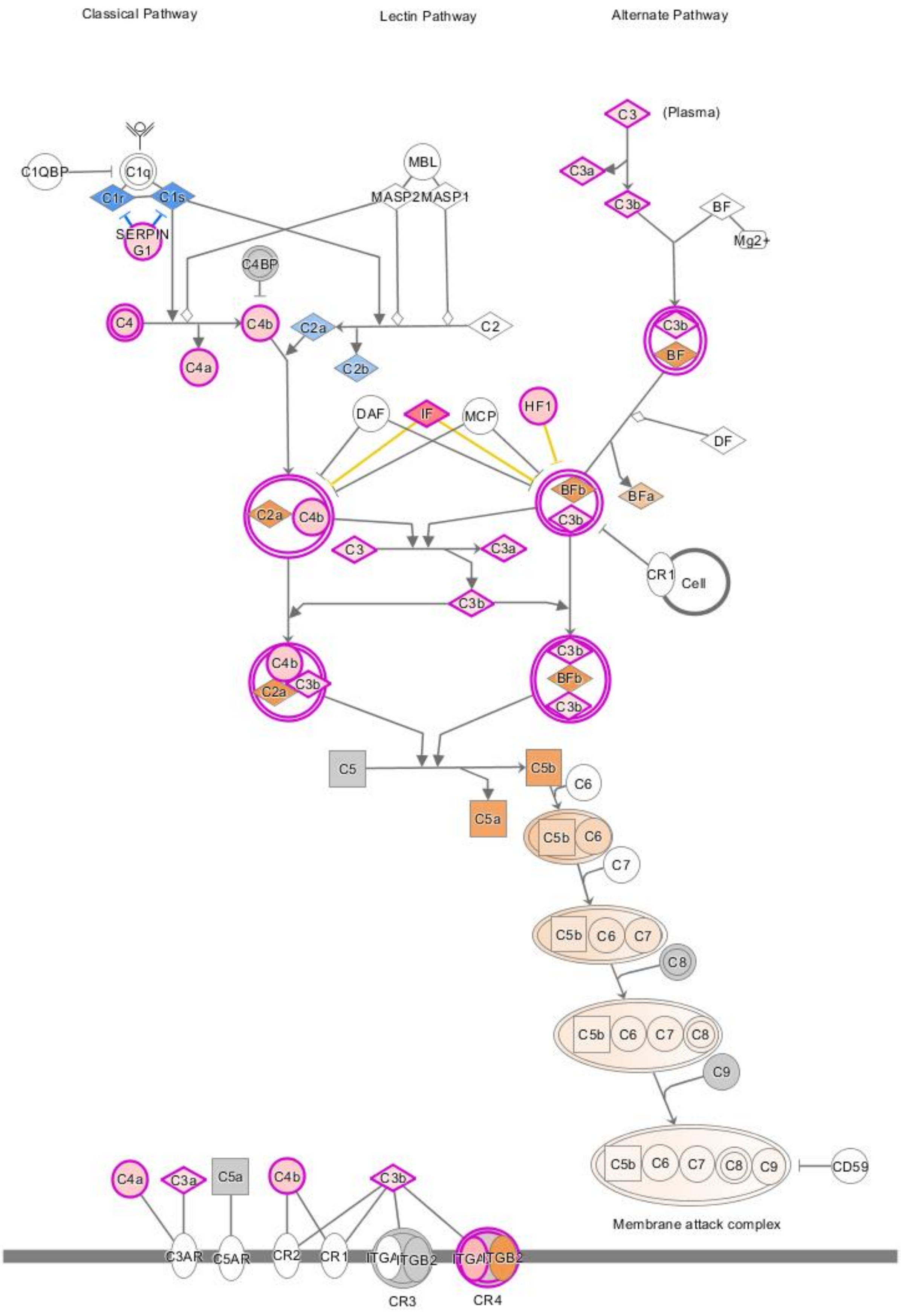

Supplement: S7 Fig — IPA bio function analysis (QIAGEN Inc., https://www.qiagenbioinformatics.com/products/ingenuity-pathway-analysis/) was carried out to assemble a network between (A) LXR/RXR activation, (B) coagulation system, (C) acute phase response signaling, (D) extrinsic prothrombin activation pathway, (E) intrinsic prothrombin activation pathway, (F) atherosclerosis signaling, and (G) complement system of the proteins differentially abundant in the AAA-high mCRP group. Red and green colors represent the measured levels of increased and decreased molecules, respectively. Orange and blue colors represent the predicted activation or inhibition of molecules or bio functions, respectively. (PDF) [file pone.0245361.s007.pdf]
