# Supplementary material for: CRP immunodeposition and proteomic analysis in abdominal aortic aneurysm
Source: PLoS One. 2021 Aug 24;16(8):e0245361. doi: 10.1371/journal.pone.0245361 (PMC8384196; doi:10.1371/journal.pone.0245361)
Supplement: S1 Table — (DOCX) [file pone.0245361.s008.docx]

**S1 Table. CRP immunostaining scores according to serum CRP levels**

|  | Serum CRP ≤ 0.1 mg/dL (n=14) | Serum CRP > 0.1 mg/dL  (n=10) | *P* value |
| --- | --- | --- | --- |
| Age, y | 68.4±7.2 | 68.3±9.4 | 0.987 |
| Male, n (%) | 12 (85.7) | 10 (100.0) | 0.618 |
| **Anti-CRP** |  |  |  |
| Immunopositivity, n (%) |  |  | 1 |
| - Negative | 1 (7.1) | 0 (0.0) |  |
| - Positive | 13 (92.9) | 10 (100.0) |  |
| Location, n (%) |  |  | 0.001 |
| - Negative | 1 (7.1) | 0 (0.0) |  |
| - Junction | 13 (92.9) | 3 (30.0) |  |
| - Diffuse | 0 (0.0) | 7 (70.0) |  |
| Atheroma immunointensity, n (%) |  |  | 0.002 |
| - [0] Negative | 1 (7.1) | 0 (0.0) |  |
| - [1+] Weak | 9 (64.3) | 3 (30.0) |  |
| - [2+] Moderate | 4 (28.6) | 0 (0.0) |  |
| - [3+] Strong | 0 (0.0) | 7 (70.0) |  |
| **Anti-mCRP** |  |  |  |
| Immunopositivity, n (%) |  |  | 1 |
| - Negative | 1 (7.1) | 0 (0.0) |  |
| - Positive | 13 (92.9) | 10 (100.0) |  |
| Location, n (%) |  |  | 0.003 |
| - Negative | 1 (7.1) | 0 (0.0) |  |
| - Junction | 13 (92.9) | 4 (40.0) |  |
| - Diffuse | 0 (0.0) | 6 (60.0) |  |
| Atheroma immunointensity, n (%) |  |  | 0.007 |
| - [0] Negative | 1 (7.1) | 0 (0.0) |  |
| - [1+] Weak | 11 (78.6) | 2 (20.0) |  |
| - [2+] Moderate | 2 (14.3) | 3 (30.0) |  |
| - [3+] Strong | 0 (0.0) | 5 (50.0) |  |
