# Supplementary material for: CRP immunodeposition and proteomic analysis in abdominal aortic aneurysm
Source: PLoS One. 2021 Aug 24;16(8):e0245361. doi: 10.1371/journal.pone.0245361 (PMC8384196; doi:10.1371/journal.pone.0245361)
Supplement: S2 Table — (DOCX) [file pone.0245361.s009.docx]

**S2 Table. Serum CRP levels and clinical characteristics in patients with abdominal aortic aneurysm**

|  | Serum CRP ≤ 0.1 mg/dL  (n=31) | Serum CRP > 0.1 mg/dL  (n=38) | *P* Value |
| --- | --- | --- | --- |
| Age, y | 67.5±7.4 | 68.4±9.2 | 0.665 |
| Male, n (%) | 27 (87.1) | 37 (97.4) | 0.242 |
| Size, cm | 5.7±1.2 | 6.5±1.5 | 0.013 |
| WBC, count/µL | 6551±1466 | 7126±1487 | 0.129 |
| Anatomic location, n (%) |  |  | 0.153 |
| - Common iliac artery | 4 (12.9) | 0 (0.0) |  |
| - Infrarenal | 24 (77.4) | 30 (78.9) |  |
| - Juxtarenal | 3 (9.7) | 7 (18.4) |  |
| Etiological, n (%) |  |  | 0.589 |
| - Degenerative | 27 (87.1) | 30 (78.9) |  |
| - Inflammatory | 1 (3.2) | 1 (2.6) |  |
| Morphological, n (%) |  |  | 0.165 |
| - Fusiform | 28 (90.3) | 28 (73.7) |  |
| - Saccular | 0 (0.0) | 2 (5.3) |  |
| Weight, kg | 66.1±8.7 | 67.8±9.8 | 0.463 |
| Height, cm | 165.5±8.5 | 166.9±4.8 | 0.407 |
| Diabetes, n (%) | 4 (13.3) | 5 (13.9) | 1.000 |
| Hypertension, n (%) | 16 (51.6) | 23 (62.2) | 0.39 |
| Tuberculosis, n (%) | 3 (10.3) | 2 (5.6) | 0.801 |
| Cardiovascular, n (%)  Disease history^a^, n (%) | 10 (38.5) | 9 (25.0) | 0.392 |
| Alcohol, n (%) | 17 (56.7) | 19 (50.0) | 0.762 |
| Smoking, n (%) | 22 (73.3) | 26 (68.4) | 0.862 |
| Dyslipidaemia^b^, n (%) | 20 (64.5) | 24 (63.2) | 0.907 |

^a^ Included history of stroke, myocardial infarction, or angina requiring percutaneous coronary intervention

**^b^** Patients who were on statin medication.
