# Supplementary material for: CRP immunodeposition and proteomic analysis in abdominal aortic aneurysm
Source: PLoS One. 2021 Aug 24;16(8):e0245361. doi: 10.1371/journal.pone.0245361 (PMC8384196; doi:10.1371/journal.pone.0245361)
Supplement: S3 Table — (DOCX) [file pone.0245361.s010.docx]

**S3 Table. Characteristics of patients included in the proteomics analysis**

| Characteristics | AAA-high CRP | AAA-low CRP | AAD |
| --- | --- | --- | --- |
|  | (n=7) | (n=3) | (n=2) |
| Age (years) | 67.4±7.8 | 62.3±3.8 | 66.0±11.3 |
| Male, n (%) | 7 (100.0) | 3 (100.0) | 0 (0.0) |
| Serum CRP level, mg/dL | 0.8±0.8 | 0.1±0.0 | 1.8±2.4 |
| Aneurysm size, cm | 6.4±1.4 | 5.8±0.3 | - |
| WBC, count/µL | 7585.7±1640 | 7200.0±1705 | 10050.0±2192 |
| Body mass index, kg/m^2^ | 23.0±4.5 | 26.1±5.0 | 23.4±1.1 |
| Diabetes mellitus, n (%) | 1 (14.3) | 1 (33.3) | 0 (0.0) |
| Hypertension, n (%) | 5 (71.4) | 3 (100.0) | 1 (50.0) |
| Previous cardiovascular disease ^a^, n (%) | 0 (0.0) | 1 (33.3) | 0 (0.0) |
| Alcohol consumption, n (%) | 2 (28.6) | 3 (100.0) | 1 (50.0) |
| Smoking, n (%) | 7 (100.0) | 2 (66.7) | 0 (0.0) |
| Dyslipidaemia ^b^, n (%) | 2 (28.6) | 2 (66.7) | 0 (0.0) |
| Arteriosclerosis obliterans, n (%) | 0 (0.0) | 1 (33.3) | 0 (0.0) |

^a^ Included history of stroke, myocardial infarction, or angina requiring percutaneous coronary intervention

^b^ Patients who were on statin medication.

AAA-high CRP indicates abdominal aortic aneurysm with strong and diffuse CRP immunopositivity; AAA-low CRP, abdominal aortic aneurysm with weak and focal CRP immunopositivity; AAD, ascending aortic dissection.
