# Supplementary material for: CRP immunodeposition and proteomic analysis in abdominal aortic aneurysm
Source: PLoS One. 2021 Aug 24;16(8):e0245361. doi: 10.1371/journal.pone.0245361 (PMC8384196; doi:10.1371/journal.pone.0245361)
Supplement: S4 Table — (DOCX) [file pone.0245361.s011.docx]

**S4 Table. Number of differentially expressed proteins in each group**

| Groups | Up | Down | Total |
| --- | --- | --- | --- |
| AAA-high CRP vs. AAD | 268 | 159 | 427 |
| AAA-low CRP vs. AAD | 347 | 316 | 663 |
| AAA-high CRP vs. AAA-low CRP | 293 | 186 | 479 |
| AAA-high CRP vs. AAA-low CRP (except AAD) | 137 | 34 | 171 |

Fold changes >2 are denoted as “Up”, and those $\leq$0.5 are denoted as “Down”, FDR <0.01
