# Supplementary material for: CRP immunodeposition and proteomic analysis in abdominal aortic aneurysm
Source: PLoS One. 2021 Aug 24;16(8):e0245361. doi: 10.1371/journal.pone.0245361 (PMC8384196; doi:10.1371/journal.pone.0245361)
Supplement: S5 Table — (DOCX) [file pone.0245361.s012.docx]

**S5 Table. Top 11 proteins differentially expressed between the AAA-high CRP group and the AAA-low CRP group after excluding the AAD group**

| Accession ID | Gene Symbol | Description | -Log Student's *t*-test *P* value | Student's *t*-test Difference | Student's *t*-test statistic |
| --- | --- | --- | --- | --- | --- |
| Q6IA69 | *Nadsyn1* | Glutamine-dependent NAD(+) synthetase | 12.4714 | 5.27136 | 2.1848 |
| P31947 | *Sfn* | 14-3-3 protein sigma | 12.1829 | 4.84685 | 2.02782 |
| P46926 | *Gnpda1* | Glucosamine-6-phosphate isomerase 1 | 11.8743 | 4.59056 | 1.92817 |
| P07476 | *Ivl* | Involucrin | 13.2292 | 4.4444 | 1.91252 |
| P26885 | *Fkbp2* | Peptidyl-prolyl cis-trans isomerase FKBP2 | 11.4093 | 4.27567 | 1.80315 |
| P36952 | *Serpinb5* | Serpin B5 | 11.036 | 4.20683 | 1.76826 |
| Q9UN86 | *G3bp2* | Ras GTPase-activating protein-binding protein 2 | 6.31179 | 4.12877 | 1.56651 |
| O75154-3 | *Rab11fip3* | Isoform 3 of Rab11 family-interacting protein 3 | 11.3862 | 3.85135 | 1.64929 |
| Q14031 | *Col4a6* | Collagen alpha-6(IV) chain | 12.6892 | 3.78399 | 1.65226 |
| P00749 | *Plau* | Urokinase-type plasminogen activator | 8.41535 | 3.562 | 1.46959 |
| P25189-2 | *Mpz* | Isoform L-MPZ of Myelin protein P0 | 10.1939 | 3.55571 | 1.51366 |
